# Effects of Resistance Training in Muscle Mass and Markers of Muscle Damage in Adults with Down Syndrome

**DOI:** 10.3390/ijerph18178996

**Published:** 2021-08-26

**Authors:** Antonio J. Diaz, Ignacio Rosety, Francisco J. Ordonez, Francisco Brenes, Natalia Garcia-Gomez, Cristina Castejon-Riber, Manuel Rosety-Rodriguez, Marco Bernardi, Jose Ramon Alvero-Cruz, Miguel A. Rosety

**Affiliations:** 1 School of Nursing, University of Cadiz, 11003 Cadiz, Spain; antoniojesus.diaz@uca.es; 2 School of Medicine, University of Cadiz, 11003 Cadiz, Spain; natalia.garciagomez@uca.es; 3 School of Sports Medicine, University of Cadiz, 11003 Cadiz, Spain; franciscojavier.ordonez@uca.es (F.J.O.); manuel.rosetyrodriguez@uca.es (M.R.-R.); 4 Puerta del Mar University Hospital, University of Cadiz, 11003 Cadiz, Spain; salud.deporte@uca.es; 5 School of Education Sciences, University of Cordoba, 14071 Cordoba, Spain; ccastejon@uco.es; 6 Department of Physiology and Pharmacology V. Erspamer, Sapienza University of Rome, 00185 Rome, Italy; marco.bernardi@uniroma1.it; 7 Department of Human Physiology, Histology, Pathological Anatomy and Sports Physical Education, University of Malaga, 29071 Malaga, Spain; alvero@uma.es; 8 School of Sports Sciences, University of Cadiz, 11003 Cadiz, Spain; miguelangel.rosety@uca.es

**Keywords:** Down syndrome, resistance training, body composition, muscle damage, creatine kinase

## Abstract

Recent studies have emphasized that regular exercise should be encouraged as a key part of care and support for people with Down syndrome (DS). However, muscle hypotonia has traditionally been considered a major barrier to resistance training (RT) in people with DS. The main objective of this study was to analyze the impact of circuit RT on markers of muscle damage. The secondary objective was to assess the influence of a RT program on body composition and work task performance. Thirty-six men with DS were recruited and randomly assigned to perform a circuit RT program with six stations 3 days/week for 12 weeks (n = 18) or to a control group (n = 18). Body composition was assessed by bioelectrical impedance analysis. Serum markers of muscle damage (creatine kinase, myoglobin, and lactate dehydrogenase) were determined at baseline and at the end of training weeks 1, 6, and 12. Work task performance was assessed using the weighted pail-carry test. RT did not induce significant changes in markers of muscle damage during the intervention. Furthermore, muscle mass and work task performance were significantly improved in the exercise group. These findings suggest that circuit RT can be used safely to increase muscle mass and work task performance in young adults with DS. Muscle hypotonia should not be considered a major barrier to exercise in people with DS, provided that qualified staff design and supervise all training sessions.

## 1. Introduction

In recent decades, the life expectancy of people with Down syndrome (DS) has increased considerably as a result of improved care and now exceeds 60 years [1]. This increase in longevity has resulted in an extended period of adulthood that may require a multidisciplinary care team to ensure long-term personal well-being and functional capacity [2]. In this respect, a recent study [3] has pointed out that people with DS are at increased risk of premature sarcopenia as indicated by lower levels of muscle mass and physical performance when compared to age-matched individuals without DS. Accordingly, the assessment of muscle mass and functional status should be included in the routine clinical management of this population starting at a young age [3,4].

For the reasons already mentioned, physical activity should be promoted, given that physical fitness is independently associated with longer survival in adults with intellectual disability (ID). In fact, improving and maintaining physical fitness by means of regular exercise should be encouraged as a key part of care and support for this population [5].

Several studies have reported that aerobic training programs designed for people with DS have improved several outcomes, including functional capacity, work task performance, oxidative damage, and low-grade systemic inflammation [6,7].

On the contrary, resistance training (RT) has received much less attention in this population despite the beneficial effects of muscle strength on functional tasks of daily living and employability in adults with DS [8,9]. In a recent study, a 12-week circuit RT program significantly reduced oxidative damage by increasing antioxidant enzyme activity in young adults with DS [10]. These findings are of considerable interest given that oxidative damage has been associated with early aging [11], insulin resistance [12], and neurodegeneration [13]. In another study, parents were encouraged to promote RT programs in children and adolescents with disabilities such as DS in view of their effects on not only physical function but also mental health [14].

However, muscle hypotonia has traditionally been considered to be a major barrier to the implementation of RT programs in people with DS [15]. It is also generally accepted that regular exercise involves an inherent risk of musculoskeletal injury that could be higher for people with ID [9]. These views may explain at least in part why RT-based intervention programs have received less attention than aerobic training programs in this population. Therefore, further studies that focus on the safety of RT for people with ID are required to increase the use of these intervention programs in clinical practice. To the best of our knowledge, no previous study has assessed the impact of RT on markers of muscle damage.

We hypothesized that an intervention program based on circuit RT could improve both muscle mass and work task performance without significantly increasing markers of muscle damage. The major objective of this study was to assess the impact of circuit RT on markers of muscle damage in the DS population. The secondary objective was to determine the influence of a RT program currently in use on body composition and work task performance in people with DS.

## 2. Materials and Methods

### 2.1. Population

Thirty-six adults with DS (mean age 28.1 ± 3.3 years) were recruited to participate in this interventional study via community support groups for people with ID. They had an intelligence quotient ranging from 60 to 69, as determined by the Stanford-Binet Scale, and were diagnosed to have mild ID. Potential study participants were excluded in the event of any of the following: (1) atlantoaxial instability; (2) congenital heart disease; (3) thyroid disease; (4) high-risk lifestyle habits (smoking or alcohol consumption); (5) participation in a training program in the 6 months prior to study entry; and (6) not completing at least 90% of the training sessions.

The statistical package GRANMO v7.12 (IMIM, BCN, Spain) was used to calculate the sample size (n = 36) with an accepted two-sided alpha risk of 0.05 and a beta risk of 0.2. We allowed for a loss to follow-up rate of 10%.

Thirty-six participants with DS were randomly assigned to an exercise group (n = 18) or a control group (n = 18) using the concealment method [16]. Those included in the control group were matched for age, sex, and body mass index (BMI) with the exercise group but did not participate in any exercise training program.

### 2.2. Intervention

The intervention consisted of a circuit RT program using weight-lifting machines 3 days per week for 12 weeks (Table 1).

The training sessions were organized in the morning, given that people with ID tend to be less active in the evenings and on weekends.

Each training session started and finished with a warm-up and cool-down period, each lasting 10 min. The RT was performed in a circular fashion at six stations: arm curl (elbow flexion), triceps extension (elbow extension), leg extension, seated row, leg curl (knee flexion), and leg press (combined hip and knee extension). The exercise sequence was designed to alternate between the upper and lower limbs. Training intensity (load) was based on the 8-repetition-maximum test for each exercise [17]. Before starting the training program, all participants in the exercise group participated in a pre-training session to become familiar with resistance exercises and the 8-repetition-maximum test for each exercise. Each training session was performed in small groups of six participants and supervised by experienced staff to ensure use of correct technique and intensity of exercise.

### 2.3. Biochemical Outcomes

Blood samples were collected from the antecubital vein into tubes containing EDTA after a 12-h fast. The samples were centrifuged at 3000 rpm for 20 min in a clinical centrifuge. The plasma was separated and stored at −80 °C until further analysis. Serum samples were analyzed using one-step sandwich immunoassays for creatine kinase activity, myoglobin concentration, and lactate dehydrogenase activity (AU5800 Plus biochemical auto-analyzer, Beckman-Coulter Inc., Brea, CA, USA) as markers of muscle damage. These outcomes were assessed in each study participant at baseline (week 0) and at the end of weeks 1, 6, and 12 (24 h after the final training session scheduled in each week).

### 2.4. Assessment of Body Composition

Body composition was assessed by bioelectrical impedance analysis (BIA; Tanita TBF521) after an overnight fast. Participants were requested not to perform any moderate or vigorous exercise for 24 h before testing and to abstain from eating or drinking for 2 h before testing. They were also asked to urinate immediately before collection of the data. Measurements were obtained while the participant was standing erect and barefoot on the analyzer’s footpads and wearing either a swimsuit or undergarments. Total body water was estimated using the equation provided by the manufacturer. The instrument was calibrated before each evaluation using known resistors.

Fat mass was determined using the equation devised by Sun et al. [18], which was designed for a large population and a broad range of BMI values. In this respect, free fat mass = −9.529 + 0.696 × (height^2^/resistance) + (0.168 × weight) + (0.016 × resistance). Fat mass was calculated as the difference between body weight and free fat mass. The intraclass correlation coefficient for fat mass was 0.98 (0.97–0.99).

Muscle mass was estimated using the equation reported by Janssen et al. [19]; namely, muscle mass (kg) = [(Ht^2^/*R* × 0.401) + (sex × 3.825) + (age × −0.071)] + 5.102, where Ht is height expressed in cm, *R* is BIA resistance in ohms (for sex, men = 1), and age is expressed in years. Similarly, the intraclass correlation coefficient for muscle mass was 0.98 (0.97–0.99). The skeletal muscle index was determined by dividing the absolute muscle mass by the square of height and expressed as kg/m^2^.

The following equation was used to calculate the body mass index: BMI = weight [kg]/height [m]^2^), expressed as kg/m^2^. Height was determined with an accuracy of 0.1 cm using a precision stadiometer. Body weight was assessed with an accuracy of 0.1 kg using an electronic balance. To determine the waist-to-hip ratio, the waist circumference and hip circumference were measured using an anthropometric tape (Holtain Ltd., Crymych, UK). Waist circumference was measured halfway between the costal edge and the iliac crest. Hip circumference was measured as the greatest circumference around the buttocks. The anthropometric measurements were obtained in accordance with the guidelines of the International Society for Advancement in Kinanthropometry (ISAK) and were carried out by the same level 3 ISAK-accredited researcher and with a technical error of measurement of less than 1% for all measures [20]. All outcomes were assessed at baseline and 72 h after the end of the intervention.

### 2.5. Nutritional Intake Record

To control for the potential confounding effect of diet, relatives were carefully instructed to avoid quantitative or qualitative variations in food intake. Furthermore, they were asked to complete a food consumption frequency questionnaire for 3 days (2 weekdays and one weekend day). Energy and nutrient intake was calculated using VD-FEN 2.1 software (Madrid, Spain) based on updated Spanish food composition tables [21]. No significant difference was found between the exercise group and the control group in terms of energy intake (1811 ± 203 kcal vs. 1786 ± 194 kcal; *p* = 0.41). Furthermore, there was no significant difference in mean daily vitamin intake between the groups (vitamin E, 9.6 ± 2.1 mg/d vs. 9.3 ± 1.9 mg/d, *p* = 0.71; vitamin C, 82.2 ± 23.7 mg/d vs. 77.4 ± 22.0 mg/d, *p* = 0.58).

### 2.6. Work Task Performance

Finally, work task performance was assessed using the weighted pail-carry test, as recommended by the American College of Sports Medicine [22]. Each participant carried two 20-L buckets each weighing 10 kg around an oblong 10-m course marked with cones in a timed fashion. The score for this test, expressed in m, was recorded as the total distance covered in 30 s. Running was not permitted for safety reasons. Furthermore, all participants (n = 36) underwent a preliminary session to familiarize themselves with the correct use of the test. This outcome was measured at baseline and at 72 h after the end of the intervention.

### 2.7. Ethics and Data Analysis

The study protocol was approved by the Institutional Ethics Committee (protocol number 29-076/2018) and complied with the Declaration of Helsinki (2013). Written informed consent was obtained from each study participant or a surrogate legal representative if necessary. The study data are expressed as the mean (standard deviation). The Shapiro–Wilk test was used to assess whether the data were normally distributed. Mean values were compared using repeated measures analysis of variance with post-hoc Bonferroni correction to account for multiple tests. The reproducibility of the weighted pail-carry test was assessed by calculating intraclass correlation coefficients using a two-way mixed-effects alpha model. For all tests, statistical significance was set at an alpha level of 0.05.

## 3. Results

There were no sport-related injuries or dropouts during the study period. The overall mean adherence rate was excellent (96%).

RT significantly improved both muscle mass (21.9 ± 4.7 kg vs. 23.4 ± 5.0 kg; *p* = 0.018) and the skeletal muscle index (8.7 ± 1.8 kg/m^2^ vs. 9.3 ± 2.1 kg/m^2^; *p* = 0.030) in the exercise group. No changes were observed in the control group (Table 2).

RT caused no significant changes in markers of muscle damage at any time during the intervention program. The results are presented in Table 3.

The weighted pail-carry test scores were also significantly improved in the intervention group (33.1 ± 5.7 m vs. 41.8 ± 6.0 m; *p* = 0.004). Test-retest reliability was strong (intraclass correlation coefficient, 0.93). By contrast, no changes were observed in the control group (34.7 ± 6.3 m vs. 34.0 ± 5.9 m; *p* = 0.099).

## 4. Discussion

The main finding in this study was the lack of a significant change in serum markers of muscle damage in the exercise group during the study period. Compared to previous studies [23,24,25], this was the first interventional study based on RT that assessed markers of muscle damage in this population. Consequently, muscle hypotonia should not be considered a major barrier to exercise in people with DS, provided that qualified staff can design and supervise all RT sessions. Fear of medical procedures in general, and needles in particular, can be a challenge to providing effective health care for people with ID [26]. Accordingly, in this study, blood samples were collected just once, 24 h after a training session, when previous papers have reported that their peaks are expected to occur [27].

The current results also suggest that RT significantly increases muscle mass in adults with DS. The improvement of muscle mass was of particular interest given that young adults with DS have shown muscle mass indices and physical performance levels similar to or lower than older adults with sarcopenia [3].

Sarcopenia is associated with an increased risk of adverse outcomes, including physical disability, institutionalization, hospitalization, and mortality in the general population [28]. Fortunately, in the present study, after completion of training, participants with DS achieved skeletal muscle index levels slightly higher than the established cutoff values for sarcopenia in the general population [29].

Similarly, Cunha et al. [30] found a significant improvement in muscle mass, assessed by dual X-ray absorptiometry, in older adults without DS who had osteosarcopenia and participated in a 12-week RT program. Furthermore, gains in muscle mass were greater in the present study than in a previous study in adults with DS reported by Coelho-Junior et al. [3]. This finding could be explained at least in part by the fact that our population sample was younger (mean age, 28.1 ± 3.3 vs. 38.4 ± 12.1 years). Indeed, the muscle mass levels reported by Coelho-Junior et al. [3] were similar to or lower than those reported for older adults with sarcopenia [28]. It should be pointed out that muscle mass was assessed using bioimpedance analysis in all of these studies [3,28] Finally, in a reliability study of BIA, Kyle et al. [31] showed that these equations worked well not only in healthy individuals but also in patients with a stable water and electrolyte balance.

On the other hand, the current RT program did not significantly reduce fat mass or fat mass distribution indices in the exercise group. Similar results were found in previous studies that focused on RT intervention programs [32,33] or mixed protocols that combined both RT and endurance training [34]. Previous studies based on endurance training [35] or whole-body vibration training [36] have also reported promising results.

Several studies have reported that RT improves muscle strength in both adults [8] and adolescents [9] with ID. However, whether this improvement transfers to improved work task performance has received less attention in the literature. As hypothesized, the current results also suggested that RT improved work task performance (weighted pail-carry test). This finding could be of particular interest for young adults with DS, as their workplace activities require physical rather than cognitive skills [25]. Furthermore, Ptomey et al. [37] reported that a mixed intervention program based on RT could have a positive effect on memory and other cognitive functions in adults with DS. In this respect, dementia has been included among the most common comorbidities in adults with DS hospitalized in Spanish internal medicine departments in the last decade [38]. On the other hand, Shields et al. [25] found no significant differences in work task performance in younger people with DS (aged 14–22 years) after completing a 10-week strength training program. These differences could be explained, at least in part, because our RT program was longer (12 vs. 10 weeks). Additionally, the intensity of training sessions was determined considering the 8-RM test for each exercise, in contrast to the 1-RM that was used by Shields et al. [25]. Lastly, the RT program reported by Shields et al. [25] was led by mentors recruited from students at a school of physiotherapy. It should be emphasized that neither sport-related injuries nor drop-outs were reported. In this regard, intervention programs based on RT should be not only effective but also safe, given that sport-related injuries and discomfort may lead participants with ID to interrupt their training, increasing drop-out rates, and sedentary lifestyles [37]. In fact, no serious adverse events have been recorded in previous studies on this topic [23,24,25]. In a more detailed way, Shields et al. [25] reported that no training sessions were missed due to soreness, injury, or illness as a result of the RT program in young people with DS. The strengths of the current study include the homogeneous and large sample size. Conversely, previous studies focused on the influence of regular exercise on people with ID recruited mixed (male and female) groups in order to increase sample size with the aim of strengthening research design [24,25,39]. In addition, previous studies have recruited participants with ID matched for intelligence quotient but with different diagnoses [8]. Furthermore, the presence of a control group consisting of adults with DS matched for age, sex, and BMI may reduce the recruitment bias of healthy controls. Lastly, the present study assessed time-course variations in several markers of muscle damage during a 12-week RT program instead of assessing them at different time points after acute bouts of exercise, as reported by previous studies [27,40]. Finally, the present study has some limitations that should be considered. Despite the use of regression equations to calculate the body composition, it should be noted that they showed high R^2^ and low standard error of the estimate values in previous studies [18,19]. We did not assess markers of muscle damage at different time points after the training session, which would have provided more information regarding the release and clearance of each biomarker in this population group. Furthermore, use of weight-lifting machines may limit the reproducibility of our study findings when exercise equipment is not available in mental health settings.

## 5. Conclusions

The presented circuit RT programme induced no significant changes in markers of muscle damage at any time during the intervention in young adults with DS. In addition, it improved both muscle mass and work task performance in the exercising group. Accordingly, well-designed and supervised resistance exercise workouts should be clearly promoted in clinical practice. Further studies with longer follow-up periods are still required to determine for how long these beneficial effects persist in this population group. Similarly, future studies focused on RT programmes that use free weights could be of great interest to guarantee its reproducibility, even at home, in case of pandemics.

## Figures and Tables

**Table 1:** Characteristics of the 12-week circuit resistance training programme, comprised of 6 stations, performed by participants in the intervention group.

Protocol	1st–2nd-wk	3rd–4th-wk	5th–6th-wk	7th–8th-wk	9th–10th-wk	11th–12th-wk
Load	40%	45%	50%	55%	60%	65%
Sets	2	2	2	2	2	2
Rep	10	10	8	8	6	6
Rest	90	90	90	90	90	90

Note: wk: Week. Load: expressed as percentage of 8 repetition-maximum (8RM) test. Rep: Number of repetitions. Rest: Resting periods between stations expressed in seconds.

**Table 2:** Influence of the circuit resistance training programme on body composition in adults with Down syndrome at baseline and after completing the intervention programme.

Characteristics	Study Group		Control Group	
	Pre-Test	Post-Test	Baseline	Final
Age (years)	28.4 (3.6)	28.4 (3.6)	27.8 (3.0)	27.8 (3.0)
Weight (kg)	71.1 (8.2)	72.1 (9.1)	69.8 (8.5)	70.2 (8.7)
BMI (kg/m^2^)	31.4 (5.7)	31.6 (6.0)	30.8 (5.2)	31.0 (5.5)
WC (cm)	91.4 (12.8)	90.8 (13.4)	88.9 (13.3)	89.0 (13.4)
WHR	0.85 (0.6)	0.86 (0.7)	0,83 (0.7)	0.84 (0.8)
FM (kg)	19.8 (8.9)	19.3 (9.2)	18.6 (9.5)	18.9 (9.7)
MM (kg)	21.9 (4.7)	23.4 (5.0) ^a,b^	20.9 (5.1)	20.6 (5.1)
SMI (kg/m^2^)	8.7 (1.8)	9.3 (2.1) ^a,b^	8.5 (1.6)	8.4 (1.5)

Note: Results are expressed as mean (sd). BMI: body mass index; WC: waist circumference; WHR: waist to hip ratio; FM: fat mass; MM: muscle mass; SMI: skeletal muscle index. ^a^ *p* < 0.05 versus pre-test; ^b^ *p* < 0.05 versus control group (final).

**Table 3:** Serum markers of muscle damage at different time points during the intervention programme based on resistance training in young adults with Down syndrome.

Markers	Study Group				Control Group			
	0-wk	1-wk	6-wk	12-wk	0-wk	1-wk	6-wk	12-wk
CK	196.4 (39.8)	201.8 (40.3)	209.3 (41.5)	211.1 (40.7)	193.7 (38.2)	195.9 (39.1)	192.1 (38.6)	194.2 (39.3)
Mb	63.1 (6.7)	64.9 (7.2)	67.3 (7.1)	68.4 (7.4)	62.8 (6.5)	62.4 (6.4)	61.3 (6.5)	62.4 (6.7)
LDH	128.0 (26.9)	131.1 (27.6)	134.2 (28.1)	135.9 (27.8)	126.5 (27.2)	127.8 (26.9)	124.6 (28.0)	125.9 (27.5)

Note: Results expressed as mean (sd). wk: week; CK: creatine kinase activity expressed as U/L; Mb: myoglobin concentration expressed as ng/mL. LDH: lactate dehydrogenase activity expressed as U/L. Changes did not reach statistical significance (*p* > 0.05).

## Data Availability

All data are available upon reasonable request to the corresponding author.
